# Systemic lupus erythematosus and antineutrocytic cytoplasmic antibody-associated vasculitis overlap syndrome presenting mainly with alveolar hemorrhage: A case report and literature review

**DOI:** 10.1097/MD.0000000000036356

**Published:** 2023-12-01

**Authors:** Siyu Yang, Jin Zhou

**Affiliations:** a School of Medical and Life Sciences, Chengdu University of Traditional Chinese Medicine, Chengdu, China; b Rheumatology Department, The Second People’s Hospital of Yibin City, Yibin, China.

**Keywords:** anti-neutrophil cytoplasmic antibody-associated vasculitis, diagnosis, SLE/AAV overlap syndrome, systemic lupus erythematosus

## Abstract

**Rationale:**

Systemic lupus erythematosus (SLE) and anti-neutrophil cytoplasmic antibody (ANCA)-associated vasculitis (AAV) are 2 different diseases that can manifest in the same person, which are known as SLE/AAV overlap syndrome. This overlap syndrome is difficult to diagnose, a high rate of missed diagnosis and misdiagnosis, and a poor prognosis.

**Patient concerns:**

A 52-year-old woman was diagnosed with SLE in 2019. She was readmitted to our hospital in October 2021 because of abdominal pain and melasma for 10 days.

**Diagnoses:**

She had positive anti-dsDNA, decreased complement C3 and C4, fever, polyarthralgia, and hemolytic anemia. She was diagnosed as microscopic polyangiitis according to the American College of Rheumatology 2022 AAV classification criteria (she had 4 items: no nasal lesions, eosinophils < 1 × 10^9^, negative c/PR3-ANCA antibodies, and positive p-ANCA antibodies. The score was 6 points).

**Interventions:**

The patient was treated with methylprednisolone 200 mg and cyclophosphamide 0.2 g immunosuppressive therapy.

**Outcomes:**

After 2 months of follow-up, the patient’s symptoms, including abdominal pain, melena, hematuria, and hemoptysis, resolved completely. And she underwent a reexamination of chest computed tomography and the results showed the previous exudation had been absorbed.

**Lessons:**

AAV should be considered in lupus patients with the above symptoms, especially the progressive decrease of hemoglobin. Relevant examinations are needed to confirm the diagnosis. Early diagnosis and accurate treatment of SLE/AAV overlap syndrome are beneficial to patients’ better prognosis and control the treatment cost.

## 1. Introduction

Systemic lupus erythematosus (SLE) is a complex systemic autoimmune disease (AD) caused by abnormal immune function.^[1]^ Multisystem and many organs may be damaged (such as skin, joints, urinary, and vascular systems). SLE will have a wide spectrum of serum autoantibodies including anti-nuclear antibody (ANA) and anti-dsDNA antibody. ANA seropositivity (ANA at a titer of ≥1:80 on HEp-2 cells) is the entry criterion of SLE.^[2]^ Anti-neutrophil cytoplasmic antibody (ANCA)-associated vasculitis (AAV) includes microscopic polyangiitis (MPA), granulomatosis with polyangiitis (Wegener), and eosinophilic granulomatosis with polyangiitis (Churg-Strauss syndrome), and its typical serological sign is that ANCAs can be detected.^[3]^ Serious organ-threatening disease involvement with rapidly progressive glomerulonephritis (GN), diffuse alveolar hemorrhage (DAH) but also gastrointestinal.^[4]^ Generally, SLE and AAV are 2 different diseases, but a case was reported that a patient can have SLE and AAV at the same time, which called SLE/AAV overlap syndrome.^[5]^ Whether SLE or MPA mainly involves the kidney, and the probability of DAH is about 9%.^[6]^ What we report a case is a 52-year-old female patient with SLE/AAV overlap syndrome diagnosed by clinical manifestations, serology, and imaging examination. It is rare for the patient to have alveolar hemorrhage as the main manifestation, while the renal function is normal. It aims to identify common factors that can better help us identify patients at risk of this complication.

## 2. Case presentation

**Figure 1.**
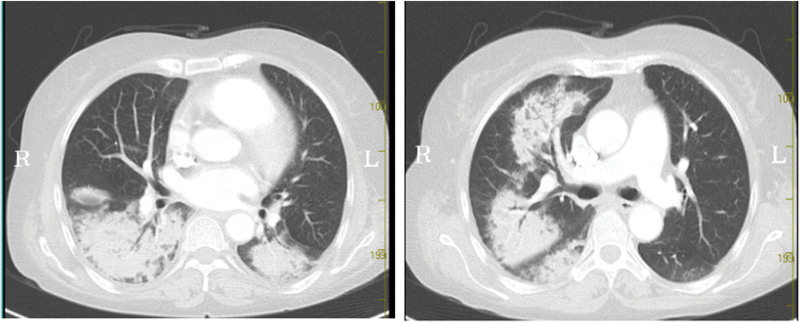
There were scattered patchy exudation and consolidation in both lungs.

**Figure 2.**
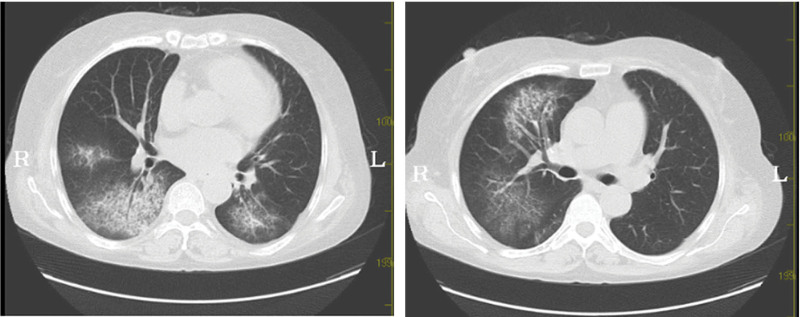
The exudation was significantly absorbed.

A 52-year-old woman was diagnosed with SLE in 2019 (she had positive anti-dsDNA, decreased complement C3 and C4, fever, polyarthralgia, and hemolytic anemia). She was readmitted to our hospital in October 2021 because of abdominal pain and melasma for 10 days. On initial physical examination, her blood pressure was 111/76 mm Hg, pulse 91, respiratory frequency 20, conscious, soft whole abdomen, epigastric tenderness, and no rebound pain. Laboratory tests showed that Coomb test was positive, with mild anemia, and occult blood in stool was also positive (Table 1). On October 10, 2021, the patient developed the symptom of coughing up bright red blood, with hemoglobin gradually decreased, and she was urgently completed with computed tomography (CT) pulmonary angiography. The results showed multiple bilateral patchy exudations are present throughout the lungs. The CT value was about 56 HU, which suggested the possibility of alveolar hemorrhage (Fig. 1). Considering that she had vasculitis, we improved 5 tests of vasculitis. The results showed that myeloperoxidase (MPO)-ANCAs were also positive (Table 1). The patient was treated with methylprednisolone 200 mg and cyclophosphamide 0.2 g immunosuppressive therapies. Methylprednisolone was adjusted to 60 mg on October 16, and cyclophosphamide was discontinued on October 18 with a total dose of 0.8 g. Chest CT reexamination on October 18 after treatment showed that multiple bilateral patchy were significantly absorbed (Fig. 2).

**Table 1:** Laboratory data

Item	Result	Unit
Thyroid function test		
FT3	2. 23	pmol/L
FT4	8. 64	pmol/L
A-TPO	320. 83	IU/mL
A-TG	41. 47	IU/mL
Autoantibody profile		
AMA-M2	+	
ds-DNA	+	
CENP-B	+	
RO-52	++	
SS-A	++	
RNP	+	
Fecal occult blood test	+++	
Complements		
C3	Normal	
C4	Normal	
Anticardiolipin antibodies	-	
ANA		
ANA titer	1:320	
Coombs test		−
p-ANCA	+	
MPO-ANCA	+	
β2-GP1-Ab	24. 53	U/mL
Blood routine examination		
Hemoglobin	105	g/L
Blood platelets		
Liver function test		
Alanine transaminase	21.4	U/L
Aspartate aminotransferase	31.6	U/L
Albumins	32.3	g/L
Kidney function test		
Creatinine	54.8	μmol/L
Glomerular filtration rate	107.44	mL/min/1.73 m^2^
Urinalysis		
Occult urinating blood	+	
Albuminuria	−	
Urine microalbumin	10	

− = negative, + = weak positive, ++ = positive, A-TG = thyroglobulin, A-TPO = thyroid peroxidase, ANA = anti-nuclear antibody, ANCA = anti-neutrophil cytoplasmic antibody, FT3 = free triiodothyronine, FT4 = free thyroxine, MPO = myeloperoxidase, SS = Sjogren's syndrome.

After a multidisciplinary meeting of rheumatology, immunology, respiratory, and radiology experts, she was diagnosed as MPA according to the American College of Rheumatology 2022 AAV classification criteria^[7]^ (she had 4 items: no nasal lesions, eosinophils < 1 × 10^9^, negative c/PR3-ANCA antibodies, and positive p-ANCA antibodies. The score was 6 points). According to the patient’s previous symptoms, signs, and auxiliary examinations, the expert team believed that this was an overlap syndrome caused by SLE/AAV, resulting in DAH. The patient’s renal function was normal, with no proteinuria, and the kidneys were not involved at this time. And the patient refused to complete the renal puncture biopsy, so this test was not completed.

**Figure 3.**
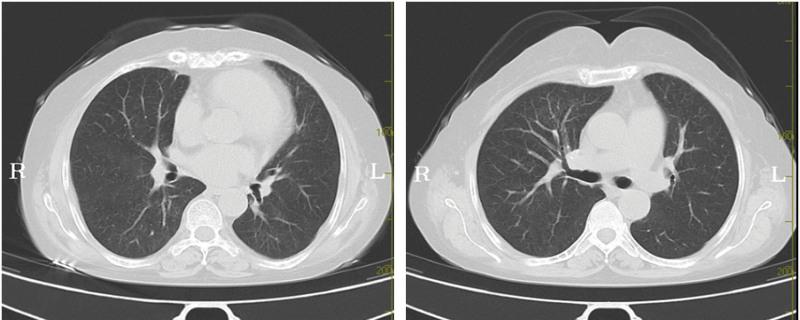
The previous exudation had been absorbed, but there were scattered patchy ground-glass density shadows and linear lesions.

In the following months of follow-up, the patient continued to be treated with prednisone and underwent cyclophosphamide pulse therapy. The patient’s symptoms, including abdominal pain, melena, hematuria, and hemoptysis, resolved completely. On November 27, 2021, she underwent a reexamination of chest CT and the results showed the previous exudation had been absorbed, but there were scattered patchy ground-glass density shadows and linear lesions, mainly involving the interstitial lung, which could not be excluded from SLE (Fig. 3).

## 3. Systematic literature review

A systematic literature review allowed the identification of 5 additional cases of SLE/AAV overlap syndrome with alveolar hemorrhage. The patients were all women. The median age at diagnosis of overlap syndrome was 52 years (range 17–74). SLE followed by AAV or MPA occurred in 3/6 patients, both occurred in 2/6 patients, and AAV occurred first in only 1 patient. Corticosteroids and immunosuppressants are the most common treatment options. Anti-MPO antibodies were detected in all patients, whereas no patient had anti-PR3 antibodies; 50% of the patients achieved remission (3/6). One patient died of cancer (hepatocellular carcinoma), and another patient died of pneumonia after 2 years of follow-up (Table 2).

**Table 2:** Literature review: patients with the SLE/AAV overlap syndrome from previous reports

Number	Author	Time (yr)	Gender	Age	Associated autoimmune disease	First disease and age at diagnosis (yr)	Second disease and age at diagnosis (yr)	MPO-ANCA	PR3-ANCA	SLEDAI	LowC3/C4	Treatment	Outcome	Renal clinical and biological involvement
1^[8,9]^	Motohide Isono	2011	F	74	Selective IgM deficiency	SLE, 74	MPA, 74	+	−		C3:0. 39 mg/mL C4:0. 01 mg/mL	CS + PE	Died	Serum creatinine 2.6 mg/dL
2^[10]^	Shoko Kobayashi	2009	F	42	–	SLE, 37	MPA, 42	+	−	4		CS	Remission	Hematuria or albuminuria
				48		SLE, 37	MPA, 42	−	−	8		CS	Died	
3^[11]^	Pierre-Andre Jarrot	2016	F	57	–	SLE, 38	AAV, 55	+	−	16	−/−	CS, CYC, HCQ	Remission	RPGN hematuria/leucocyturia
4^[11]^	Pierre-Andre Jarrot	2016	F	29	Graves–Basedow disease	AAV, 12	SLE, 17	+	−	17	−/−	CS, CYC	Remission	Proteinuria hematuria/leucocyturia
5^[11]^	Pierre-Andre Jarrot	2016	F	74	Idiopathic thrombopenic purpura	SLE, 74	AAV, 74	+	−	26	−/−	PE, CS, CYC, MMF	Died	RPGN hematuria/leucocyturia

AAV = ANCA-associated vasculitis, ANCA = anti-neutrophil cytoplasmic antibody, CS = corticosteroids, CYC = cyclophosphamide, HCQ = hydroxychloroquine, MMF = mycophenolate mofetil, MPO = myeloperoxidase, PE = plasma exchange, RPGN = rapid progressive glomerulonephritis, SLE = systemic lupus erythematosus, SLEDAI = SLE disease activity index.

## 4. Discussion

SLE and AAV have almost no similarities in clinical manifestations, and rarely appear together. SLE and AAV can be distinguished according to their own antibody spectrum, histopathological characteristics, and demographic characteristics.^[12]^ However, some patients showed mixed clinical patterns, which met the diagnostic criteria of SLE and AAV. Jarrot et al^[11]^ found that SLE associated with AAV may be a group of diseases called SLE/AAV overlapping syndrome. In multiple autoimmune syndrome (MAS) with SLE background, autoimmune thyroid disease (AITD), Sjogren's syndrome, and antiphospholipid antibody syndrome were most common, whereas vasculitis was rare.^[13]^ Martin-Nares et al^[14]^ estimated the frequency of overlap between AAV and ADs. Among 247 patients with AAV, 28 of 247 (11. 3%) were complicated with other systemic AD, of which only 2 cases (7%) were SLE. SLE/AAV overlap syndrome is a new diagnosis, and there are few cases recorded in the literature, and there is no strictly defined standard. This overlap syndrome is difficult to diagnose, a high rate of missed diagnosis and misdiagnosis, and a poor prognosis.

In this case, after 2 years of diagnosis of SLE, this patient was hospitalized because of “abdominal pain and black stools.” Our initial consideration is that the digestive tract is damaged because of her lupus flare. It is reported that SLE can involve multisystem, of which 40% to 60% of SLE patients can affect gastrointestinal tract.^[15]^ However, further examination showed that the patient’s SLE disease activity index (SLEDAI) score was only 2, and his complement was normal, which was not consistent with the flare of SLE. When SLE patients are accompanied by hematuria, abdominal pain, melena, and hemoptysis, it is not necessarily a recurrence of lupus. According to the patient’s symptoms, such as hemoptysis and progressive decrease of hemoglobin, she was improved by 5 tests of vasculitis and CT pulmonary angiography. The patient’s symptomatology, as well as the associated imaging studies and positivity for MPO-ANCAs, supported the initial diagnosis of AAV. This case seems to meet the existing diagnostic criteria for SLE/AAV overlap syndrome. So AAV should be considered in lupus patients with the above symptoms, especially the progressive decrease of hemoglobin. Relevant examinations are needed to confirm the diagnosis. Early diagnosis and accurate treatment of SLE/AAV overlap syndrome are beneficial to patients’ better prognosis and control the treatment cost.

It is worth noting that the kidney is usually the most commonly involved organ in SLE and AAV, and about 50% SLE patients will affect the kidney.^[16]^ Renal involvement is the main clinical feature in MPA, and about 80% to 100% patients with MPA have renal involvement.^[17]^ Patients from previous reports in the literature have presented with renal clinical and biological involvement. In this case, although the patient is unwilling to improve the renal biopsy, there is no related renal injury such as proteinuria, so it is considered that the kidney is not involved this time. It is rare that patients with SLE/AAV overlap syndrome have normal renal function but instead present with DAH as the main symptom.

The precision medicine of overlap syndrome is expected. At present, glucocorticoids, cyclophosphamide, and plasmapheresis were considered as a first-line treatment option in induction treatment.^[8,9]^ Rituximab can also be used for remission induction and is not inferior to daily cyclophosphamide in inducing remission of severe AAV.^[18]^ The treatment regimens for the 6 patients in this study also revolved around hormones, cyclophosphamide, and plasmapheresis.

In this patient, SLE had been diagnosed in 2019. The patient’s disease activity was scored by SLEDAI, and the patient total score was 2 (only positive anti-dsDNA). The SLEDAI score of the 6 cases mentioned in this paper ranges from 2 to 26, which is quite different. Therefore, disease activity in patients with SLE is not associated with the occurrence of overlap syndrome, but further analysis with a larger scope is needed. This case tentatively suggests that the possibility of concurrent overlap syndrome also needs to be taken into account in the stable or mildly active phase of SLE for the diagnosis of SLE preceded by SLE/AAV overlap syndrome.

The majority of SLE/AAV occurs in female patients. Hervier et al^[5]^ analyzed 29 cases of SLE/AAV overlap syndrome, of which 11 patients with known sex were all female, and the sex was not mentioned in the remaining 18 cases. In our study, all patients are female. ADs are characterized by female bias. Sex hormones can affect the pathogenesis and gene expression of ADs. The combination of environmental factors and susceptibility loci is a significant factor promoting female SLE disease.^[19]^ SLE overlapping AAV may be induced by multiple factors, and whether it is related to disease duration, comorbidities, and infections, in addition to activity and gender, needs to be verified in large-scale studies.

Polyautoimmunity may be one of the reasons for this. This patient is known as overlapping syndrome because there are 2 ADs (SLE and AAV) at the same time, while patients with 3 or more ADs are called MAS. Rojas-Villarraga et al’s study^[13]^ included 226 patients with MAS found that AITD is the most common comorbid AD. The patient was also diagnosed with AITD, based on a positive test for thyroid peroxidase antibodies and a positive test for thyroglobulin antibodies. In this systematic review 3/5 patients had a third AD. It is emphasized that polyautoimmunity may be a mechanism for the occurrence of overlap syndrome. ADs are a group of heterogeneous diseases. They have similar subphenotypes, including signs and symptoms, nonspecific autoantibodies.^[20]^ This fact is called “the autoimmune tautology.”^[21]^ The best clinical evidence of this tautology is polyautoimmunity, which refers to the occurrence of multiple ADs in a patient. Both MAS and overlap syndrome belong to this category. Although polyautoimmunity is common in SLE, the overlapping syndrome of SLE and AAV is rare.^[22]^

The presence of neutrophil extracellular traps (NETs) may be another cause of overlap syndrome. After neutrophil activation, the target antigens of ANCA are transported from the cell cytoplasm to the membrane surface, and the Fab2 segment of ANCA can bind to the corresponding target antigens, triggering local inflammation.^[23]^ At the same time, the activation of neutrophils will form NETs. Excessive production of NETs or reduced clearance of NETs will lead to excessive exposure of target antigens such as MPO and PR3, thus forming a vicious circle. NETs also contain a variety of antigens. In addition to MPO and PR3, dsDNA, cathelicidin, HMGB1, and other antigens closely related to SLE are also present in NETs.^[24]^

In conclusion, the cases of SLE with MPA are not coincidences. If vasculitis is suspected in SLE patients, ANCA test should be performed as soon as possible, and tissue biopsy should be performed if necessary. The renal histopathology may help to distinguish the diseases. SLE GN often shows immune complex deposition on the glomerular basement membrane. In contrast, renal involvement of AAV is typically a pauci-immune crescentic GN. Early diagnosis of the patient can enable precision medicine, improved prediction, and increased survival. It has been shown that this overlapping syndrome may have a SHARED genetic background and IMMUNE pathogenesis, and future therapeutic research should be directed toward these common mechanisms, leading to new and potential treatment options. Finally, for patients with complex conditions, a multidisciplinary meeting is also an important treatment modality.

## Author contributions

**Data curation:** Siyu Yang, Jin Zhou.

**Funding acquisition:** Jin Zhou.

**Supervision:** Jin Zhou.

**Writing – original draft:** Siyu Yang.

**Writing – review & editing:** Siyu Yang, Jin Zhou.
